# Report to the State Society by Committee on Rheumatism

**Published:** 1858-05

**Authors:** Samuel De Bois


### *Report to the State Society by Committee on Rheumatism.*

By Samuel De Bois, M. D.

To make this Report as full of interest as it were possible, your Committee sought aid, by early appointing as auxiliary members several gentlemen well qualified to render assistance, but unfortunately they, in every instance, failed to make their contributions;—therefore, coming from such a source, and unaided, the inferiority of the production will give no surprise. But, justice to ourself requires it to be said that circumstances have almost wholly deprived us of the necessary time and opportunity for consultation of others.

To form a just conception of what is expected is, perhaps, the greatest difficulty connected with the duty with which this association has thought proper to honor us; for the almost universal tendency in man is to *overreach*, especially in efforts to be brought before the public. That it is not a Treatise on Rheumatism that is here called for, the fullness of the works extant, coming from men of the highest talents, abundantly argues; but, supposing it to be, to simply set forth what observation and experience have led us to believe to be some of the best established facts in relation to the nature of the disease and its best mode of treatment, and in the absence of something better and more appropriate, we submit to you the following:

New discoveries we can claim none; but in that part of our State in which we have been acquainted, Rheumatism has appeared to be on the increase with the improvement of the country, with an increasing tendency to the acute form for the last few years; and from opportunities afforded for the basis of an opinion, would say: If there is, among the diseases with which the human body is afflicted, one which is the result of *altered chemical conditions,* it is Rheumatism—all its phenomena pointing to a poison contained in the system, somehow introduced, or elaborated within, or, what is more probable, consisting of effete material which has failed to be conveyed out of the system, composed of one or more elements, the result of certain elective affinities, and possessing as some have supposed, an acid re-action, from the prevalence of acids in the excretion, and the nature of the remedies that have proved most successful. These views seem to be supported by the fact of the supervention of the complaint in those persons inhabiting climates where the variations of temperature, called changes of weather, are sudden and frequent, and especially in those whose exposure to such vicissitudes (in combination with moisture) have the effect of suddenly modifying the amount of excretions of the body, particularly that of the skin.

A general acquaintance with what has been said by our Standard Authors upon the disease rendering unnecessary a full description, we will only notice a few main points of interest.

For consideration, it is usually divided into Acute and Chronic, or Inflammatory, and Non-Inflammatory, but we see all grades of intensity of action, from the high and peculiar inflammation down to the most trifling local manifestation.

Besides its migratory characteristics, the great point of difference to be noticed between rheumatic and the ordinary inflammation is, that we never see the former running to suppuration, or to several other results common to the latter, of equal intensity. True, it is claimed by some that they have witnessed rheumatic inflammation of such intensity that suppuration was produced; but it is probable that this effect in the cases referred to was due to an intercurrent inflammation of the erysipelatous, or common variety, or that the occurrence of a deep-seated abscess, as in the hip-joint, was mistaken for Rheumatism—a case of which came under our own observation, in which the deception was truly marvelous, yet in time well cleared away. Following the distinction a little farther, as says one of our first auhorities, "Throughout all this febrile disturbance, there is no coma, no marked trouble of the stomach or of the bowels, no vomiting, no diarrhœa, no petechiæ, no aphthæ, no sordes about the mouth, all of which are of ordinary occurrence in the course of common continued fevers"; which it is fortunate to remember in some masked cases of difficult diagnosis.

There seems to be a little difference of opinion among writers, as to whether the Acute form is apt to run into the Chronic.* Our own limited observation leads to the belief that such a transition may frequently be *expected, even under active treatment.* And here, we beg leave, in few words, to give a case.

Young L., on a bright warm Sunday, in March, A. D. 1856, amused himself by wading in water running over the ice, following the course of a stream connecting two small lakes for the distance of nearly a mile; then spent some time on the ice. In a few days he was attacked with Acute Rheumatism; his pulse about 120 to 130. The usual alterative and alkaline treatment assisted by opium, was resorted to, which considerably controlled the symptoms for several weeks, but with little real improvement. Fibrous deposits, and the consequent enlargement of the joints, occurred, followed by usual maifestation of the chronic form of the complaint, when he fell into the hands of a Quack of the Thompsonian species, and is to this day a cripple.

The marked contrast observed in symptoms, and in the kind of treatment indicated in the two forms of the complaint, has tempted some into the belief that they were really two diseases; but we regard such differences only as results growing out of the same philosophical grounds, as that which discovers as striking a difference in the symptoms and treatment called for in the sthenic and asthenic forms of ordinary inflammation. Another important view of the subject may be taken by distinguishing it as *Articular, Visceral, or Neuralgic.* Mixed cases are of every day occurrence; which is sometimes a fortunate circumstance, as a means of correct diagnosis, which would in some cases be otherwise difficult.

The first point of interest in purely *Articular* is the chronic enlargement-deformity, and consequent failure of function of the joints, which frequently occurs, and *sometimes* in patients under vigorous and orthodox treatment. Of this, we meet with two kinds: one, which is the result of fibrinous deposit, the other of synovial effusion; in both the diseased action is, at first, exterior to the synovial cavity. The former we meet with most freqeuntly in the robust and plethoric, with more frequent tendency to attacking the heart, while the latter oftener is seen in those of weak constitutions, sometimes approaching a cachexia

Another lesion attendant on the more chronic cases, is that peculiar state of the synovial cavities and sheaths of the tendons which produces

---

* See Miller's Principles of Surgery, 4th American Edition, page 61; Also, Watson. Then Dunglison's Medical Dictionary, 9th Edition, Revised, on Rheumatism.

the friction-sound, a peculiar squeaking both to be heard and felt. This may, however, often be discovered in the sub-acute form of ordinary inflammation of these structures, resulting from violence.

Among internal organs liable to be attacked, we notice the heart and its appendages, the diaphragm and pleura (constituting Pleuritic Rheumatism, or Rheumatic Pleurisy), the ligamentous attachments of the liver and the stomach (constituting Gastric Rheumatism, or Rheumatic Gastralgia.)

If delirium occur in a case of Acute Rheumatism, almost certainly the heart is implicated; which it is well to remember, at least so far as to lead to such exammination of the case as to not allow this fearful complication to escape detection; for it may here be remarked, that if pericarditis once establish itself, and the patient temporarily escape with his life, the organ usually undergoes lesions that are, in time, certainly fatal. "With this exception" says WATSON, "we do not find patients in Acute Rheumatism delirious." But we have observed that patients who are apparently laboring under a strong tendency *towards* an implication of the heart in the disease, or in whom the heart is almost imperceptibly affected, appear as though inspired with a strange horror of some thing, they know not what, and somewhat resembling what has been described by patients laboring under hydrophobia, but to a minor degree, and yet seem to recover without the usual phenomena attending rheumatic pericarditis.

The *Pleuritic* we have known to so closely resemble ordinary pleurisy as to render the case puzzling to our limited training in diagnosis, at the attack. It is quite frequently confounded, no doubt, with an attack of the intercostal muscles, and oftener accompanied by the same. A peculiarity of the disease in this locality is, that, when carefully sought for, the friction-sound may be discovered, although sometimes only when the patient forcibly elevates the ribs by deep inspiration—indicating the state of a portion of the pleural surfaces. And we have sometimes witnessed sudden and violent traumatic attacks of the complaint in the pleura and intercostals, following the puncture of abscess in the mammæ, etc. The acute and continual pain that is often experienced, with the great stricture in breathing in this form of attack, is little short of that which is characteristic of true pleurisy in its severest form; and when it is of a more chronic character, the pain in the chest is of such a peculiar character, that some hypochondriac and hysterical grannies of both sexes have tried in vain, with their "pain in the chest," year after year, to die with "consumption," even down to a good old age. And, when extending to the appendages of the liver, it constitutes a large share of the

"*liver complaint*," now so polite an affection, affording Quacks an opportunity to try their skill with mandrake pill; and they are generally successful, in time—so far as the pocket is concerned.

Rheumatic Gastralgia is one sort of, or rather is frequently taken for, dyspepsia; and is often an intractable complaint, the more so from being so frequently misunderstood.

And this brings us to another boundary of the subject, viz, the *Neuralgic* form; which is, according to our observation, most usually combined with anæmia, and the consequent peculiar irritability of the nervous system; difficult to distinguish from ordinary neuralgias, if indeed there be a difference; exceedingly painful, at night especially; affecting the teeth, jaws, face, etc., and, as above said, the stomach. And here we beg liberty to offer the conjecture that *angina pectoris* with all the discovered structural or organic changes in and about the heart, will yet be found to be due to the chronic lurkings of the poison; also that fearful tetanus;—both the eccentric offspring of the rheumatic diathesis, strange as it may appear.

We also see Rheumatism fixing itself in the most aggravated form, upon those whose constitutions have undergone a decided depression from the effects of mercury or syphilis, producing the most horrible distortion of the frame—as WATSON says, "one crooked joint is the copy of its fellow." A striking instance of this was the case of Mr. B., an early settler in this State—strong and healthy—after using an astonishing quantity of *Ung Hydrarg.* by way of a general friction, for what he supposed to be itch; following its specific effects, he was attacked almost immediately with Rheumatism, which soon transformed him into the most dreadfully misshapen cripple for life.

Thus we have here a disease, appearing to occupy an intermediate place, insensibly blending with the febrile or inflammatory on one extreme, and with the cachectic on the other.

When we consider that few diseases are capable of inflicting greater amount of suffering, within a given length of time, than Rheumatism, it becomes exceedingly desirable to arrive at a knowledge of the most ready means of relief. But says one of our most reliable authors—"Now you may be sure—when men's opinions concerning the treatment of a disease which is of common occurrence, and easy recognition, are thus unsettled and diverse—you may be sure, first that no specific for that disease has yet been discovered, and secondly, that the disease is not very steadily obedient, to any remedial plan." This was uttered several years ago, and to-day we do not feel very well prepared to contradict it, but from observation and testimony, naturally infer that class of remedies which may be more directly addressed to the peculiar chemical condition of the system under the disease, will be most successful. And such experience proves to be true, besides a strictly chemical view.—The treatment is therefore *antiphlogistic. alterative*, and *restorative*.

Among the *Antiphlogistics* the means that have proved most useful in Rheumatism are, first, those of direct depletion, as bleeding, purging and emetics; secondly, sedatives, as opium, antimony, veratrum and colchicum in large doses. The *Alteratives* are mercury, the alkaline salts, iodide of potassium, colchicum, and counter-irritation (the word "alterative" being used in this paper in the sense of including the agents of the direct chemical action). The *Restoratives* are, iron, quinine, iodide of potassium, and *warmth*.

Referring again to the standard works for the details of treatment, we merely notice certain points of particular interest; and, first, with regard to *Bleeding*. Some authors recommend bleeding with considerable freedom; others, with great caution. The latter class receive our most hearty approbation. And WATSON (whose views of this disease as a whole please us best of any we have seen), after recommending the practice with some freedom, says: "Yet, looking to my own practice in Acute Rheumatism, I find that although I am in the almost daily habit of treating it,—for it, is a disorder from which our wards are never perhaps entirely free,—I rarely prescribe phlebotomy." And, again: "So that the cases in which I am tempted to employ the lancet, are few and far between." The *rule* we would here offer is: In cases of highly inflammatory re-action, as shown by the pulse, and if the patient be young and plethoric, *bleed*, and not otherwise, even in the cardiac complication, unless early in the case and in all cases with the greatest caution; for in some patients the cardiac affection will be produced by injudicious bleeding, when it would not otherwise have occurred. And in the cases which promise most from bleeding, if it be carried to the extent (by error of judgment with the practitioner, or peculiarity of the patient) of producing any thing approaching what may be called the hæmorrhagic pulse, almost certainly the patient is lost; for if the heart be too much emptied of its contents, a greater task is imposed upon it, in the greater effort required in the performance of its function. Then, when bleeding is resorted to, let it be conducted with a view to producing the greatest sedative effect, with the least loss of blood, yet guarding against actual syncope, as favoring the deposit of one of those little mischievous appendages about the valves, from the fibrinous state of the blood, with all its dreadful consequences, endocarditis being so often coincident.—Admitting the temporary relief to be usually immediate and great, and the greater facility with which other remedies act, yet we regard a tolerably full heart (other things being equal) of the two, the most favorable to the desired result of this fearful complication.

In addition to bleeding, judiciously employed, we most heartily endorse the sentiment of WATSON, in saying that *Mercury*, boldly pushed to its defibrinating effect, with *Opium*, is the only hope, after pericarditis is once fairly established. It is then that the matter becomes that of a choice between two evils,—mercurialization, or death of the patient more or less immediately: and it is then that the class of *Quacks* who plead "No Mercury," become most ridiculous, as there is no other article that will answer as well. And in the less urgent cases, the more cautious use of mercury with opium is the most valuable means in the treatment of all cases of the inflammatory form, to allay the violent action then to be superseded by the milder alteratives or eliminatives.

Pretty free *Purging*, or at least an active state of the bowels, is very essential in assisting to eliminate the poison and reduce the febrile excitement. As a sort of temporary cure, any thing that produces a great sedative or relaxing effect on the system is generally highly successful, at least in allaying the more acute symptoms; therefore we frequently find that an antimonial emetic, will produce very beneficial results; also veratrum viride, or, what seems to act still better, veratrum and colchicum equal parts, given once in three hours in such doses as to produce a great sedative effect; or the colchicum alone in large, or we might as well say, dangerous doses, as we found in one two instances in which we prescribed it, in the early part of our practice, and being a little deceived as to the strength of the article, the doses given producing some of its specific effects, such as protracted vomiting, but the Rheumatism, which was of a high grade of violence, was speedily missing, on which we took occasion to congratulate ourselves, yet secretly confessing the remedy almost as bad as the disease. This plan of operation is understood by some aside from our Profession. The Thompsonians accomplish the same by giving the lobelia inflata, in large quantities. And too good to be lost, is the expression of a gray-headed specimen of this sect, who, on hearing the above case related, and evidently ignorant of the remedy used, put on a sapient look, and gravely remarked, *"I should have paralyzed her* [the patient] *with Lobelia!"*

The colchicum in small doses, as ordinarily given, probably assists, but more especially in gout, by promoting the elimination of urea from the system.

What has been said of the merits of the alkaline salts, we believe experience will justify; and are inclined to the belief that most of the apparent failures with these remedies (at least in our practice) has been attributable to their not having been given in sufficiently large doses, and *sufficiently continued*; for almost frightful quantities seem often to be required—not grains, but drachms and ounces.

*Counter-irritation* is most particularly beneficial in the rheumatic affections of the internal organs. A patient is attacked with what his friends suppose to be a pleurisy of violent character; but, from some peculiarities, we infer the difficulty to be rheumatic, and apply a blister to the chest, when suddenly all the stricture is relieved, plainly distinguishing it from real pleurisy. Thus blistering over the stomach and liver, in rheumatic cases, often does great things in a short time—in this respect differing from the effect in inflammatory cases.

And this introduces the subject of *External Applications* in general. In the inflammatory form, patients are apt to think cold applications agreeable and beneficial; but we do not hesitate to say that they are, not only dangerous, but hurtful, favoring metastasis, and even hindering the most speedy disposition of the case. The best external application with which we are acquainted, is a hot alkaline solution, made from the wood ashes always at hand in the fireplace. These fomentations are to be large, and continued, and the results have often suggested the idea of a local specific, or chemical effect, from the potash. The more chronic cases that generally call for stimulants and frictions, may be treated externally by the rubefacient effect of strong ammoniated liniments.

In the *Neuralgic* forms, accompanied by anæmia and scrofula, ammonia must be mostly substituted for the salts of stronger base, and iron sesq. ox. used freely and *protractedly* with iodide of potassium, and, above all things with those of rheumatic habit, *extra warm clothing* of wool. Morphia will often be required for immediate relief.

And after we have said all, we sometimes find ourselves most rascally mortified for want of success, in a given case—at any rate patients will take it out in grunting, if for no other reason than to dampen the feelings of the physician in expectation of his fee.

These fragments of thought are respectfully submitted to your consideration. They are thus brief, and scattered, for want of greater facilities, and because given at random, and especially because we would avoid, as far as possible, unnecessarily repeating what has been already said by others, and therefore uncalled for; and if we have not the ability to carry it out, we would at least indorse the sentiment—

> "Floriferis ut apes in saltibus omnia libant
> Omnia nos itidem."

[*Peninsular & Independent.*

